# Impact of CYP2C19 point-of-care testing on the clinical outcome in patients receiving personalized clopidogrel therapy: systemic review and meta-analysis

**DOI:** 10.3389/fphar.2025.1621327

**Published:** 2025-11-21

**Authors:** Shaban Mohammed, Zainab Ali, Nermin Mohammed, Ayman El-Menyar, Jassim Al Suwaidi, Moza Al-Hail, Wadha Al-Muftah, Rania Abdel-Latif, Maw Shin Sim

**Affiliations:** 1 Pharmacy Department, Hamad Medical Corporation, Doha, Qatar; 2 Department of Pharmaceutical Life Sciences, Universiti Malaya, Kuala Lumpur, Malaysia; 3 Trauma and Vascular Research and Development at Hamad medical corporation, Doha, Qatar; 4 Clinical Medicine Department, Weill Cornell Medical College, Doha, Qatar; 5 Department Hamad Medical Corporation, Cardiology and Cardiovascular Surgery, Doha, Qatar; 6 Qatar Genome Program, Qatar Precision Health Institute, Qatar Foundation, Doha, Qatar; 7 Department of Pharmacology and Toxicology, Faculty of Pharmacy, Minia University, Minia, Egypt

**Keywords:** CYP2C19 point-of-care genotyping, antiplatelet therapy, percutaneous CoronaryIntervention, acute coronary syndrome, major adverse cardiovascular events

## Abstract

**Objective:**

Evidence on the utility of CYP2C19 point-of-care (POC) testing to guide antiplatelet therapy selection in patients with acute coronary syndrome (ACS) or stable coronary artery disease (CAD) undergoing percutaneous coronary intervention (PCI) is currently limited. To address this gap, a meta-analysis was conducted to assess the clinical impact of CYP2C19 POC genotyping in ACS patients treated with P2Y12 inhibitors in PCI settings. The study compared clinical outcomes between standard care and genotype-guided antiplatelet therapy in ACS or CAD patients undergoing PCI, leveraging POC genotyping for rapid therapy optimization.

**Method:**

PubMed, EMBASE, Cochrane, Scopus and ProQuest. Central databases were searched up to 30 August 2025, for studies evaluating the use of point-of-care CYP2C19 genotyping to guide antiplatelet therapy in ACS/CAD patients undergoing PCI, comparing clinical efficacy and safety with conventional P2Y12 inhibitors. Two independent reviewers assessed study eligibility, extracted data, and evaluated the risk of bias. Risk ratios (RRs) with 95% confidence intervals were computed using random-effects models, with study heterogeneity assessed by the I^2^ index. The primary outcome included major adverse cardiovascular events including myocardial infarction, stroke, stent thrombosis or death and bleeding risk within 12 months of PCI.

**Results:**

A total of four randomized controlled trials (RCTs) were included in the meta-analysis, comprising 5912 antiplatelet-treated ACS/CAD patients undergoing PCI. The analysis showed minimal statistical heterogeneity and low risk of bias. Compared with the standard treatment group, the genotype-guided group demonstrated a significantly lower risk of recurrent myocardial infarction (RR 0.54, 95% CI 0.38–0.77, P = 0.001). Although there were no significant differences in the efficacy outcomes for cardiovascular death, stroke, stent thrombosis, or bleeding complications, the calculated composite MACEs were significantly reduced in the genotype-guided group (RR 0.59, 95% CI 0.48–0.72, P = 0.001).

**Conclusion:**

Genotype-guided antiplatelet therapy using CYP2C19 POC genotyping prior to PCI in ACS/CAD patients may reduce the risk of recurrent myocardial infarction and composite MACEs compared to standard treatment, highlighting the importance of POC genotyping for facilitating rapid and effective therapeutic decision-making.

**Systematic Review Registration:**

https://www.crd.york.ac.uk/PROSPERO/view/CRD420251157778, identifier CRD420251157778.

## 1 Introduction

Clopidogrel is a widely used antiplatelet agent essential for preventing cardiovascular events, particularly in patients with acute coronary syndrome (ACS) or stable coronary artery disease (CAD) and undergoing Percutaneous Coronary Intervention (PCI) (Patti et al., 2020). However, clopidogrel’s efficacy and safety are significantly influenced by individual variability in drug metabolism, primarily mediated by the cytochrome P450 2C19 (CYP2C19) enzyme (Shuldiner et al., 2009). The metabolism of clopidogrel to its active form is a complex process involving several steps, and genetic polymorphisms in the CYP2C19 gene have been identified as key determinants of clopidogrel’s therapeutic outcomes (Brown and Pereira, 2018). Patients with certain CYP2C19 loss-of-function alleles (LOF), such as *2 and *3, exhibit reduced conversion of clopidogrel to its active metabolite, leading to decreased platelet inhibition and an increased risk of adverse cardiovascular events, including stent thrombosis and myocardial infarction (Simon et al., 2009; C et al., 2009). Given the significant impact of CYP2C19 polymorphisms on clopidogrel response, there has been growing interest in developing and implementing point-of-care (POC) testing for CYP2C19 genotyping (Mega et al., 2009). Such testing enables rapid identification of patients with CYP2C19 LOF, allowing for timely adjustment for personalized antiplatelet therapy (Cavallari et al., 2018). Patients identified as poor metabolizers can be prescribed alternative antiplatelet agents, such as prasugrel or ticagrelor, which are not dependent on CYP2C19 for activation, potentially reducing the risk of adverse cardiovascular outcomes (Trenk et al., 2008).

The clinical utility of CYP2C19 POC testing has been the subject of several studies, with varying results regarding its impact on cardiovascular and bleeding outcomes in patients treated with clopidogrel. While some studies have demonstrated that CYP2C19 testing is associated with improved cardiovascular outcomes and reduced bleeding complications, others have failed to show significant benefits, leading to ongoing debate in the field (Tsukahara et al., 2010; Klein et al., 2018; Zeb et al., 2018). The variability in study outcomes may be attributed to differences in study design, patient populations, clinical settings, and the specific genetic testing methods employed (Russmann et al., 2021; Wallentin et al., 2010). It is hypothesized that the readily availability of genetic information at the time of antiplatelet prescription may significantly impact clinical outcomes (Cavallari et al., 2018; Barrett and Topol, 2012). Immediate access of CYP2C19 genotyping results allows for direct use in clinical decision-making, whereas delays of even a week or more can hinder optimal treatment, especially in the acute phase following stent placement. Supporting this, the American Heart Association’s recent statement underscores the critical importance of rapid genetic testing to enhance the effectiveness of pharmacogenetics (PGx, the study of how genetic variation influences drug response)-guided therapy for patients with ACS or undergoing PCI (Pereira et al., 2024). Due to the limited data available on the impact of CYP2C19 genotyping during the early phase post-ACS, a comprehensive meta-analysis is necessary to systematically evaluate the effect of POC-genotyping on clopidogrel-related cardiovascular and bleeding outcomes in the acute ACS setting. This meta-analysis aims to synthesize data from existing studies to provide a clearer understanding of the clinical impact of CYP2C19 POC testing, identify potential sources of heterogeneity, and offer insights into the utility of personalized antiplatelet therapy guided by CYP2C19 genotyping.

In this meta-analysis, we will assess the overall effect of CYP2C19 POC testing on major cardiovascular events (MACE), including myocardial infarction, stroke, stent thrombosis, or death as well as bleeding associated with clopidogrel therapy. By examining these outcomes, we aim to determine whether CYP2C19 POC testing should be integrated into routine clinical practice for patients undergoing clopidogrel therapy and to what extent it can improve patient outcomes. The findings of this meta-analysis will contribute to the growing body of evidence supporting precision medicine approaches in cardiovascular care and inform clinical guidelines on using CYP2C19 genotyping in optimizing antiplatelet therapy.

## 2 Methods

### 2.1 Search strategy

A comprehensive search of PubMed, EMBASE, Cochrane Central, Scopus and ProQuest databases from 1 January 1996, to 30 August 2025, was conducted to identify studies comparing genotyped guided strategy use vs. the conventional P2Y12 inhibitor use in patients with ACS or stable CAD undergoing PCI. The key words used were “clopidogrel’’, “CYP2C19’’, “PCI”, “Spartan”, “Verigene”, “GMEX” or “point of care” with the limits of human and English language. To look for additional relevant publications, we further performed a hand-search of reference list of the selected studies.

### 2.2 Selection criteria and data extraction

We identified studies eligible for further review by performing initial screen of identified titles or abstracts. The following inclusion criteria were used: 1) prospective or randomized control trials including patients undergoing PCI, 2) studies comparing genotyped guided versus conventional P2Y12 inhibitor non-genotyped use based on CYP2C19 genetic testing using a point of care CYP2C19 assay, 3) studies reporting outcomes of interest which were MACE, defined as myocardial infarction, stroke, stent thrombosis or death) and bleeding risk within 12 months of PCI.

Two independent reviewers (ZA and NM) screened all titles and abstracts retrieved from the database search. Full-text articles were then assessed for eligibility against the predefined inclusion and exclusion criteria. Discrepancies between reviewers were resolved through discussion; if consensus could not be reached, a third reviewer (SM) adjudicated.

For data extraction, four authors (ZA, NM, SM and RA) independently extracted relevant information using a standardized data extraction form, which included study characteristics and risk of bias assessments. Extracted data were cross-checked for accuracy, and any inconsistencies were clarified through consensus. The protocol for this systematic review was prospectively registered in the PROSPERO database (Registration ID: CRD420251157778).

**FIGURE 1:**
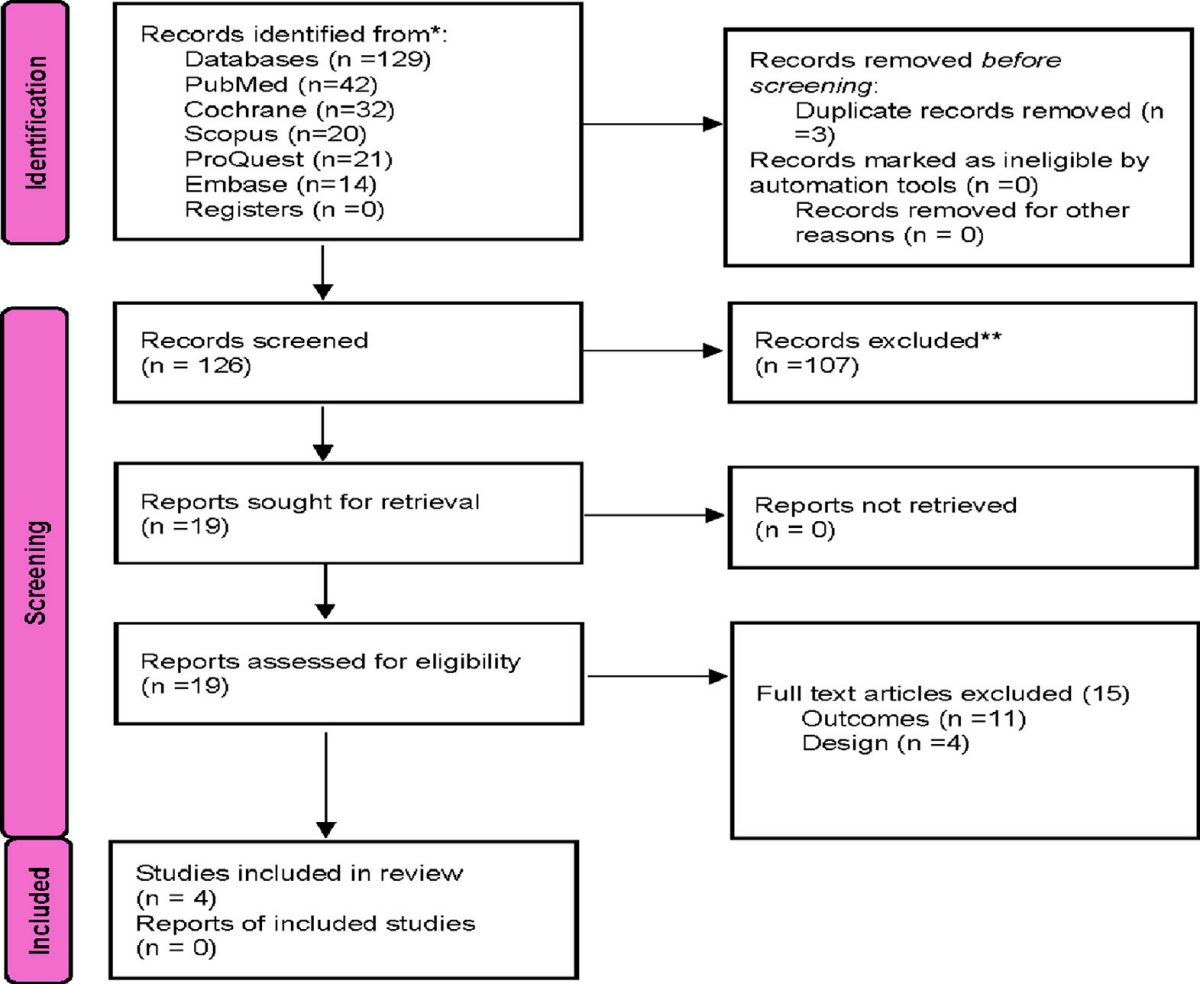
Flow diagram of the systematic review process.

This systematic review and meta-analysis were conducted and reported using the Preferred Reporting Items for Systematic Reviews and Meta-Analyses (PRISMA) statement as described in Figure 1. A total of 19 studies were identified as potentially relevant and screened for inclusion in the study. Then, a second screening based on full text review was performed. Studies comparing genotyped guided strategy use vs. conventional P2Y12 inhibitor use in patients with ACS or stable CAD undergoing PCI were only included in the final analysis (Tables 1, 2). Studies that did not match the outcomes of interest were excluded.

**TABLE 1:** Characteristics of studies included in the systematic review and meta-analysis.

Source	Sample size	Arms	Antiplatelet treatment	Endpoint	Age (mean (SD) (years)	Gender Women %	Diabetes %	HTN %	Smoker %
TAILOR - PCI (2020) (Pereira et al., 2020)	1849	**Genotyped group** CYP2C19*2 or *3 LOF alleles received ticagrelor Genotyped as noncarriers or inconclusive results received clopidogrel (n = 903) **Conventional group** received conventional therapy as randamized (n = 946)	Of the 903 patients assigned to genotype-guided therapy, 764 (85%) received ticagrelor. In contrast, 932 out of 946 patients (99%) assigned to conventional therapy were administered clopidogrel	The primary end point was a composite of cardiovascular death, myocardial infarction, stroke, stent thrombosis, and severe recurrent ischemia at 12 months	62	25	28	62	25
POPular genetics (2019) (Claassens et al., 2019)	2,488	**Genotyped group** as CYP2C19*2 or *3 LOF alleles received ticagrelor or prasugrel genotyped as noncarriers received clopidogrel (n = 1242 **Standard -treatment group** received P2Y12 antiplatelet therapy as a standard care (n = 1246	In the genotype-guided group, 60.6% of patients received clopidogrel, and 39.1% were prescribed ticagrelor or prasugrel following the availability of genetic results. In the standard-treatment group, 92.7% of patients received clopidogel, while 7.0% were treated with ticagrelor or prasugrel	The two primary outcomes were net adverse clinical events defined as death from any cause, myocardial infarction, definite stent thrombosis, stroke, or major bleeding defined according to Platelet Inhibition and Patient Outcomes (PLATO) criteria at 12 months	62	25.5	12	41	46
PHARMCLO trial (2018) (Notarangelo et al., 2018)	888	**Pharmacogenomic arm** received genotyping antiplatelet guided therapy (n = 448) **Standard of care arm** received P2Y12 as a standard care (n = 440)	150 out of 448 were CYP2C19 LOF carriers, 225 received either ticagrelor or prasugrel while 194 (43.3%) received clopidogrel. In standard treatment arm 50.7% received clopidogrel while 181 patients received prasugrel or ticagrelor	primary composite endpoint of cardiovascular death and the first occurrence of nonfatal myocardial infarction, nonfatal stroke, and major bleeding defined according to Bleeding Academic Research Consortium type 3 to 5 criteria	<70	32	26.5	74.3	22.5
Bedside testing of CYP2C19 (2021) (Al-Rubaish et al., 2021)	687	**Standard group** received clopidogrel (n = 312) **Genotyped groups** were genotyped for CYP2C19 loss-of-function alleles and carriers were prescribed ticagrelor and noncarriers were prescribed clopidogrel (n = 375)	31 patients in the genotype-guided group received ticagrelor, while all other patients in both groups were treated with clopidogrel	combined ischemic and bleeding outcome, comprising myocardial infarction, non-fatal stroke, cardiovascular death, or Platelet Inhibition and Patient Outcomes major bleeding 1 year after STEMI.	55	0	89	83	48

**TABLE 2:** Summary of included studies reporting CYP2C19 genotype and allele frequencies.

Study (year)	Country/region (ethnicity)	Variants assayed	Genotype frequencies (as reported)	Allele frequencies (as reported or derivable)	HWE
**TAILOR-PCI (2020)**	Multinational (US, Canada, South Korea, Mexico; mixed ethnicity)	CYP2C19*2 (rs4244285), CYP2C19*3 (rs4986893) (LOF); CYP2C19*17 (rs12248560) (GOF, *post hoc*)	*1/*1 ≈ 55–60% *1/*2 or *1/*3 ≈ 28–30% *2/*2 or *2/*3 ≈ 5–6% *1/*17, *17/*17, *2/*17 ≈ 30%	LOF = (2 + 3) = 0.194	χ^2^ = 0.06, p ≈ 0.8
**POPular Genetics (2019)**	Netherlands/Belgium/Italy (predominantly European)	CYP2C19*2 (rs4244285), CYP2C19*3 (rs4986893) (LOF)	*1/*1 ≈ 55–60% *1/*2 or *1/*3 ≈ 28–30% *2/*2 or *2/*3 ≈ 5–6% *1/*17, *17/*17, *2/*17 ≈ 30%	*1 = 0.828 *2 = 0.170 *3 = 0.00245	χ^2^ = 2.2, p ≈ 0.53
**PHARMCLO (2018)**	Italy; European ancestry required per protocol	CYP2C19*2 (rs4244285) (LOF), CYP2C19*17 (rs12248560) (GOF)	*1/*2 = 29.2% *2/*2 = 4.3% *1/*17 = 31.3% *17/*17 = 7.8%	*2 = 0.189 *17 = 0.235	χ^2^ = 1.99, p ≈ 0.16
**“Bedside testing of CYP2C19” (Al-Rubaish 2021)**	Saudi Arabia (Middle Eastern/Arab)	CYP2C19*1, CYP2C19*2 (rs4244285), (LOF), CYP2C19*17 (rs12248560) (GOF), CYP2C19*4 (rs28399504) (rare)	*1/*1 = 40.3% *1/*2 = 14.5% *2/*2 = 0.5% *1/*17 = 30.4% *2/*17 = 7.0% *17/*17 = 7.0% *1/*4 = 0.5%	*1 = 0.629 *2 = 0.112 *17 = 0.257 *4 = 0.0025	χ^2^ = 1.97 p ≈ 0.92

LOF, Loss-of-function allele (*2, *3), GOF, Gain-of-function allele (*17), HWE, Hardy–Weinberg equilibrium, χ^2^ = Chi-square statistic, p = p-value.

### 2.3 Data extraction and quality assessment

The screening phase was performed for the extraction of the baseline characteristics of each trial, study quality, and outcomes of interest. Data was crosschecked for any errors during data extraction. The methodological quality of genetic association studies was assessed using the Q-Genie tool, which is specifically designed to evaluate the validity of genetic association studies across 11 domains. Each domain is scored on a 7-point Likert scale ranging from poor (1) to excellent (7). The domains assess the clarity of study rationale, outcome definition, comparability of comparison groups, technical and non-technical classification of exposure, potential sources of bias, adequacy of sample size and power, appropriateness of analytic methods, reporting of power and multiple comparisons, completeness of results reporting, and interpretation of findings. Scores from each domain were summed to generate a total score for each study, with higher scores indicating stronger methodological quality (Sohani et al., 2015). The risk of bias in the included randomized controlled trials (RCTs) was assessed using the Cochrane Risk of Bias (RoB 2.0) tool. This tool evaluates five key domains: (1) bias arising from the randomization process, (2) bias due to deviations from intended interventions, (3) bias due to missing outcome data, (4) bias in measurement of the outcome, and (5) bias in selection of the reported result. Each domain was rated as low risk of bias, no information, or high risk of bias. Assessments were conducted independently by two reviewers (ZA and NM), with disagreements resolved through discussion and consensus.

### 2.4 Statistical analysis

A meta-analysis was conducted to assess the impact of POC testing CYP2C19 LOF gene polymorphisms on clopidogrel treatment outcomes in ACS or CAD patients undergoing PCI. The included studies were randomized controlled trials comparing CYP2C19-guided versus standard therapy.

Statistical analysis was performed using Stata 18.1, employing random-effect model for independent cardiovascular and bleeding events. was applied to estimate pooled risk ratios (RRs) with corresponding 95% confidence intervals (CIs) for independent outcomes. Composite outcomes were derived by pooling data for cardiovascular death, myocardial infarction, stroke, and stent thrombosis as reported by individual trials.

Heterogeneity was assessed using Cochran’s Q test (P < 0.10 indicating significance) and the τ^2^ test to evaluate variance. The I^2^ test measured the percentage of variation due to heterogeneity, with values ≥ 50% indicating significant heterogeneity. The significance of RRs was determined using the Z-test.

Publication bias was also evaluated using a funnel plot and quantitatively using Egger’s regression asymmetry test, in which the standard error of each study was plotted against its log odds ratio. Symmetry in the distribution of studies around the pooled effect estimate was visually inspected to detect potential bias. In addition, the pseudo 95% confidence limits were superimposed to aid in assessing the presence of small-study effects. Statistical significance was defined as P < 0.05 (two-sided).

## 3 Results

### 3.1 Characteristics of the included studies

The study selection process for inclusion in the meta-analysis, following PRISMA guidelines, is outlined in Figure 1. A total of 173 unique citations were identified through the database search, of which 16 were deemed potentially eligible. Ultimately, 4 studies were included, comprising 5912 ACS or CAD patients undergoing PCI who met the inclusion criteria (Pereira et al., 2020; Al-Rubaish et al., 2020; Claassens et al., 2019; Notarangelo et al., 2018). All included studies were RCTs. Among the 2,968 patients in the genotyping-guided group, CYP2C19 POC testing was performed, and antiplatelet therapy was administered based on the genotyping results. Of these, 1431 patients received either ticagrelor or prasugrel, while 1537 received clopidogrel. In the standard care group, 2,944 patients received antiplatelet therapy according to routine practice, with 92.4% (2,721 patients) receiving clopidogrel, and only 223 patients were prescribed alternative antiplatelet therapy.

All studies included in the analysis focus on composite outcomes that combine cardiovascular and bleeding events over a 12-month period. Common endpoints across all studies are cardiovascular death, myocardial infarction, and stroke. Stent thrombosis is part of the primary endpoint in two studies (Pereira et al., 2020; Claassens et al., 2019). Major bleeding is also a consistent endpoint, though defined using different criteria: the PLATO criteria are used in the Bedside and POPULAR trials (Al-Rubaish et al., 2020; Claassens et al., 2019), while the BARC type 3–5 criteria are applied in the PHARMCLO study (Notarangelo et al., 2018). The primary composite outcome in most studies includes cardiovascular death, myocardial infarction, and stroke, with the POPULAR trial (Claassens et al., 2019) uniquely considering death from any cause, whereas other studies focus solely on cardiovascular death.

### 3.2 Quality assessment

The Q-Genie assessment demonstrated that all four included studies achieved high overall quality scores, ranging from 56 to 63 out of a maximum of 77. Most domains, including study rationale, outcome definition, sample size, analytic methods, and interpretation of results, were consistently rated as excellent (scores of 6–7). However, a key limitation across all studies was the technical classification of the exposure, with each scoring only 1 point, reflecting limited reporting of laboratory genotyping methods and error rates. Additionally, the domain of reporting of results scored 0 across all studies, indicating a lack of full transparency in presenting genotype frequencies and effect estimates. Despite these gaps, the overall Q-Genie scores indicate that the studies were of generally high methodological quality, suitable for inclusion in the review (Table 3).

**TABLE 3:** Q-Genie quality assessment scores of included genetic association studies.

Domain no.	Domain	Description	Scoring 1–7 (poor – excellent) study title			
			TAILOR PCI	PoPular genetics	PHARMCLO trail	Bedside testing of CYP2C19
1	Rationale for study	Clarity and justification for conducting the genetic association study	7	7	7	7
2	Selection and definition of outcome of interest	Definition and measurement of the clinical or intermediate phenotype (e.g., MACE, HPR)	7	7	7	7
3	Selection and comparability of comparison groups	Appropriateness and comparability of control/comparator group; minimisation of selection bias	6	7	3	6
4	Technical classification of the exposure	Genotyping methods, laboratory quality control, error rate reporting	1	1	1	1
5	Non-technical classification of the exposure	Definition of genetic variant, allele, and genotype categorisation; use of accepted nomenclature	7	7	7	7
6	Other sources of bias	Consideration of population stratification, misclassification, or other design biases	6	6	6	6
7	Sample size and power	Adequacy of sample size and reporting of statistical power calculations	7	7	4	7
8	Analytic methods	Appropriateness of statistical models, adjustment for confounders, handling of multiple comparisons	7	7	7	7
9	Statistical power and multiple comparisons	Explicit reporting of statistical power and correction for multiple testing	7	7	7	7
10	Reporting of results	Completeness and transparency in reporting genotype frequencies, effect estimates, and CIs	0	0	0	0
11	Interpretation of results	Appropriateness of conclusions; consideration of study limitations, biological plausibility, and consistency with existing evidence	7	7	7	7
Total score			62	63	56	62

**Scoring Interpretation: ≥45 points**: High quality; **35–44 points**: Moderate quality; **<35 points**: Low quality; **0**: Not applicable.

### 3.3 Assessment of publication bias

Most studies demonstrated adequate random sequence generation and low risk of attrition or reporting bias (Table 4). However, common limitations included high risk in blinding of participants and personnel across PHARMACLO (2018), POPULAR Genetics (2019), and TAILOR-PCI (2020). Detection bias due to outcome assessment was judged as high in PHARMACLO (2018), TAILOR-PCI (2020), and the Bedside Testing study (2021). The Bedside Testing study additionally showed high risk under “other bias,” while PHARMACLO was rated high for both performance and detection bias. Overall, while the included trials were generally methodologically sound, the lack of consistent blinding procedures remains a recurrent source of potential bias.

**TABLE 4:** Risk of bias assessment of included randomized controlled trials.

Study	Random sequence generation (selection bias)	Allocation concealment (selection bias)	Blinding of participants and personnel (performance bias)	Blinding of outcome assessment (detection bias)	Incomplete outcome data (attrition bias)	Selective reporting (reporting bias)	Other bias
**PHARMACLO Trial, 2018**	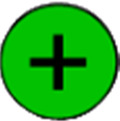	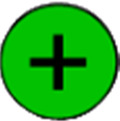	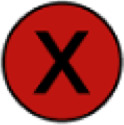	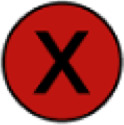	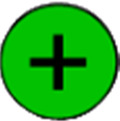	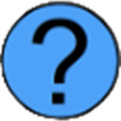	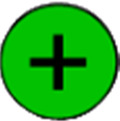
**POPULAR Genetics, 2019**	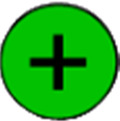	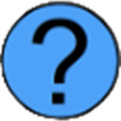	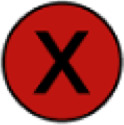	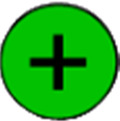	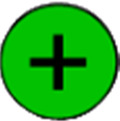	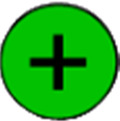	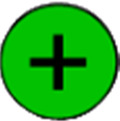
**TAILOR-PCI, 2020**	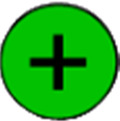	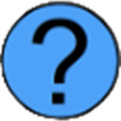	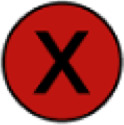	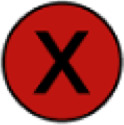	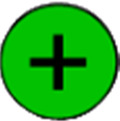	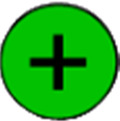	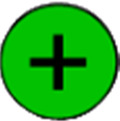
**Bedside testing of CYP2C19 vs. conventional clopidogrel, 2021**	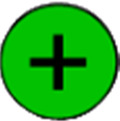	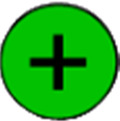	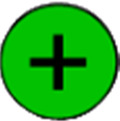	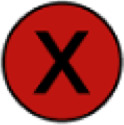	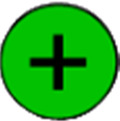	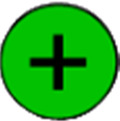	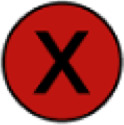

Judgement: 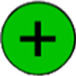 Low; 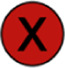
high; 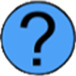 No information.

**FIGURE 2:**
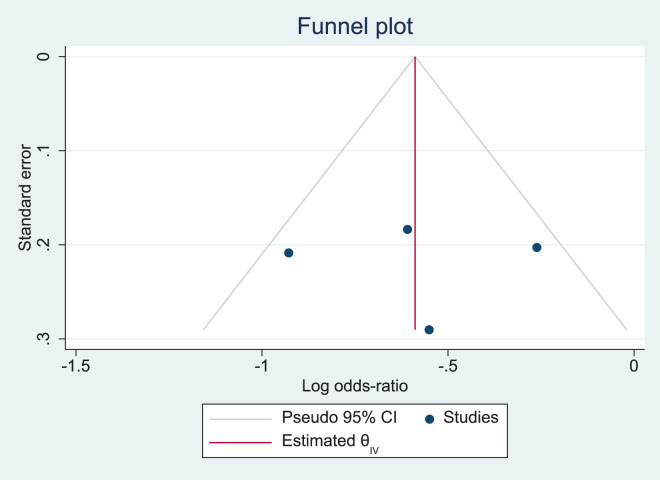
Funnel plot assessing publication bias.

The funnel plot (Figure 2) demonstrated an approximately symmetrical distribution of the included studies around the pooled log odds ratio, suggesting no clear evidence of publication bias. All studies fell within the pseudo 95% confidence limits, further supporting the absence of small-study effects. However, given the relatively small number of studies, the interpretation of funnel plot asymmetry remains limited.

### 3.4 The impact of CYP2C19 POC genetic testing on the cardiovascular death

**FIGURE 3:**
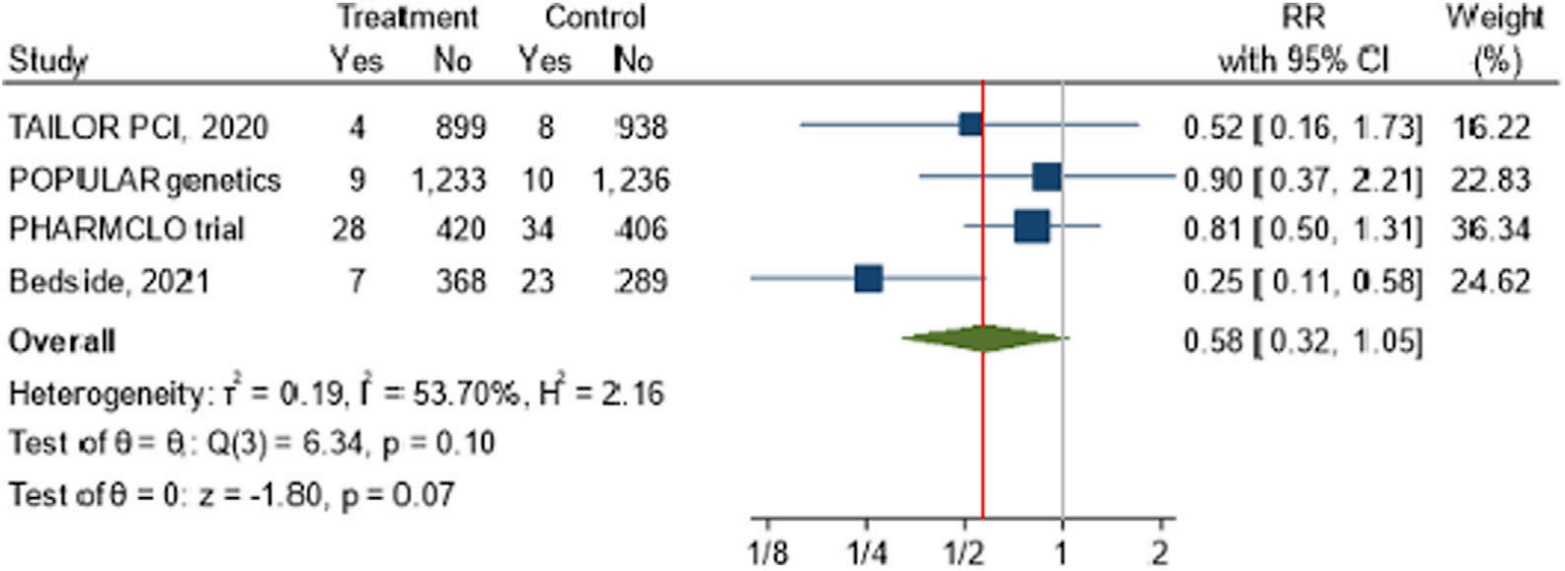
The impact of CYP2C19 point-of-care (POC) genotyping-guided antiplatelet therapy on cardiovascular death in patients undergoing percutaneous coronary intervention (PCI).

A meta-analysis was conducted to assess the impact of POC PGx-guided therapy on cardiovascular death across four RCTs: TAILOR PCI, POPular Genetics, PHARMCLO Trial, Bedside testing of CYP2C19 (2021). The overall pooled RR for cardiovascular death in the treatment group, compared to the control group, was 0.58 (95% CI: 0.32, 1.05), as illustrated in the forest plot Figure 3. The pooled analysis from these four studies indicates a 42% reduction in the risk of cardiovascular death in the treatment group (RR = 0.58). The overall p-value of 0.07 from the z-test for the combined effect suggests a trend toward reduced cardiovascular mortality, but this result did not reach conventional statistical significance. Moderate heterogeneity was detected across the studies, with an I^2^ value of 53.70% and τ^2^ = 0.19.

### 3.5 The impact of CYP2C19 POC genetic testing on the recurrent myocardial infarction

**FIGURE 4:**
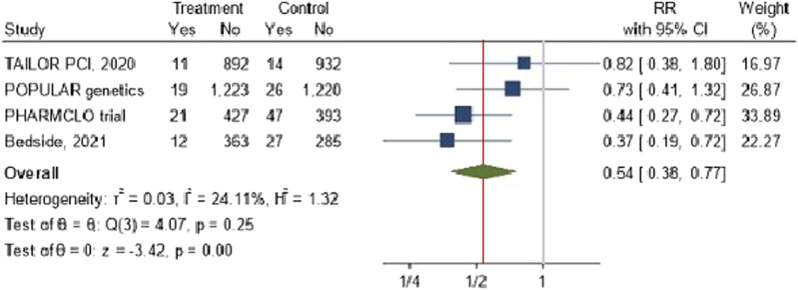
The impact of CYP2C19 point-of-care (POC) genotyping-guided antiplatelet therapy on myocardial infarction in patients undergoing percutaneous coronary intervention (PCI).

The forest plot Figure 4 illustrates the pooled analysis of four studies investigating the effect of POC PGx-guided therapy on the incidence of recurrent myocardial infarction (MI). The overall risk ratio across the studies indicates a significant reduction in recurrent MI in the treatment group compared to the control group, with a RR of 0.54 (95% CI: 0.38, 0.77). The effect appears consistent across studies with minimal heterogeneity was observed among the included studies, with an I^2^ value of 24.11% and τ^2^ = 0.03, particularly in trials such as PHARMCLO and Bedside (2021), which showed a robust benefit.

### 3.6 The impact of CYP2C19 POC genetic testing on stroke

**FIGURE 5:**
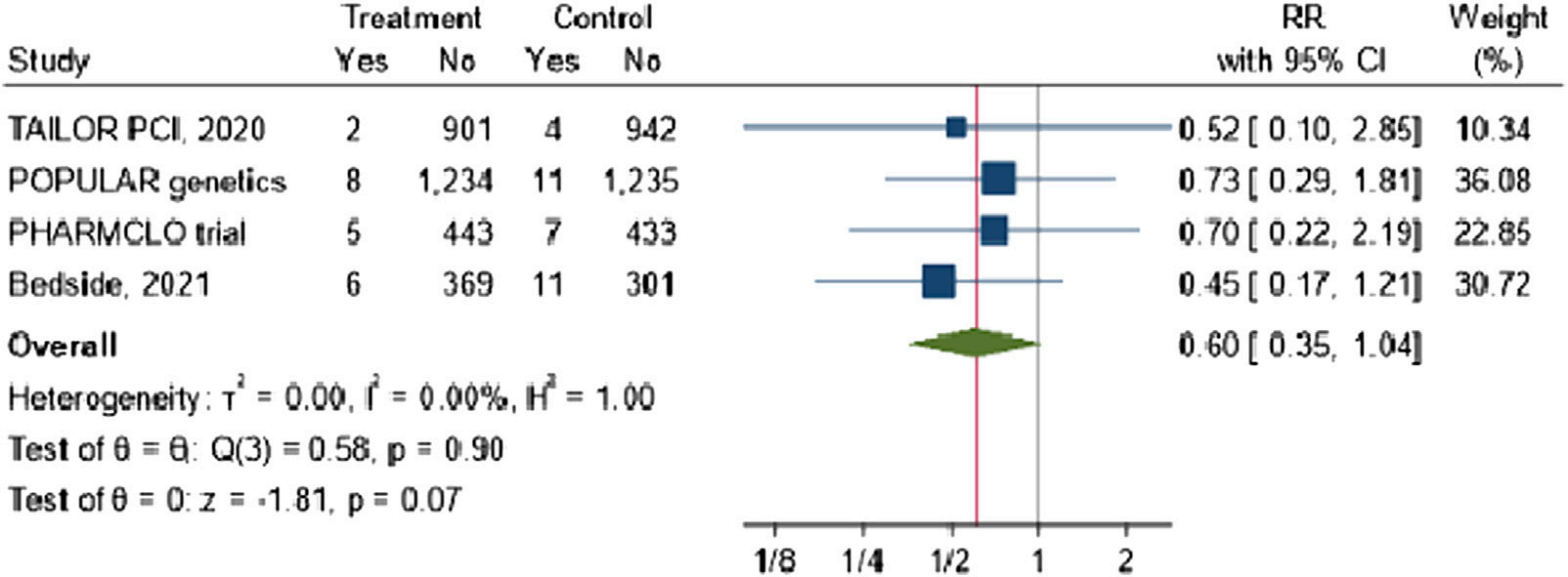
The impact of CYP2C19 point-of-care (POC) genotyping-guided antiplatelet therapy on stroke in patients undergoing percutaneous coronary intervention (PCI).

The combined RR across the four studies was 0.60 (95% CI: 0.35, 1.04), suggesting a 40% potential reduction in stroke risk with no heterogeneity was observed across the included studies, as evidenced by an I^2^ value of 0%. However, the result was not statistically significant (p-value = 0.07) Figure 5.

### 3.7 The impact of CYP2C19 POC genetic testing on stent thrombosis

**FIGURE 6:**
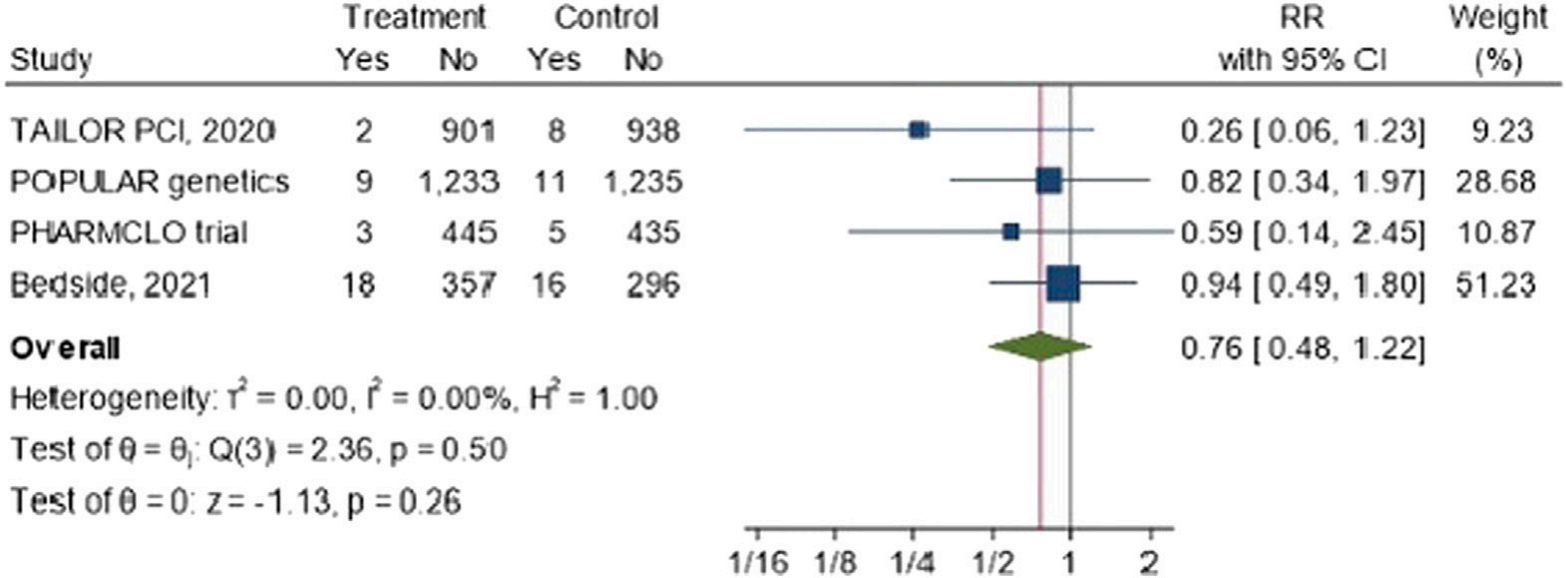
The impact of CYP2C19 point-of-care (POC) genotyping-guided antiplatelet therapy on stent thrombosis in patients undergoing percutaneous coronary intervention (PCI).

The forest plot Figure 6 shows the overall risk ratio for stent thrombosis across the four studies is 0.76 (95% CI: 0.48, 1.22). The results suggested that there is a reduction risk of stent thrombosis of 26%. However, the results are not statistically significant, and the confidence intervals are wide, particularly in studies like TAILOR PCI and PHARMCLO Trial.

### 3.8 The impact of CYP2C19 POC genetic testing on bleeding complications

**FIGURE 7:**
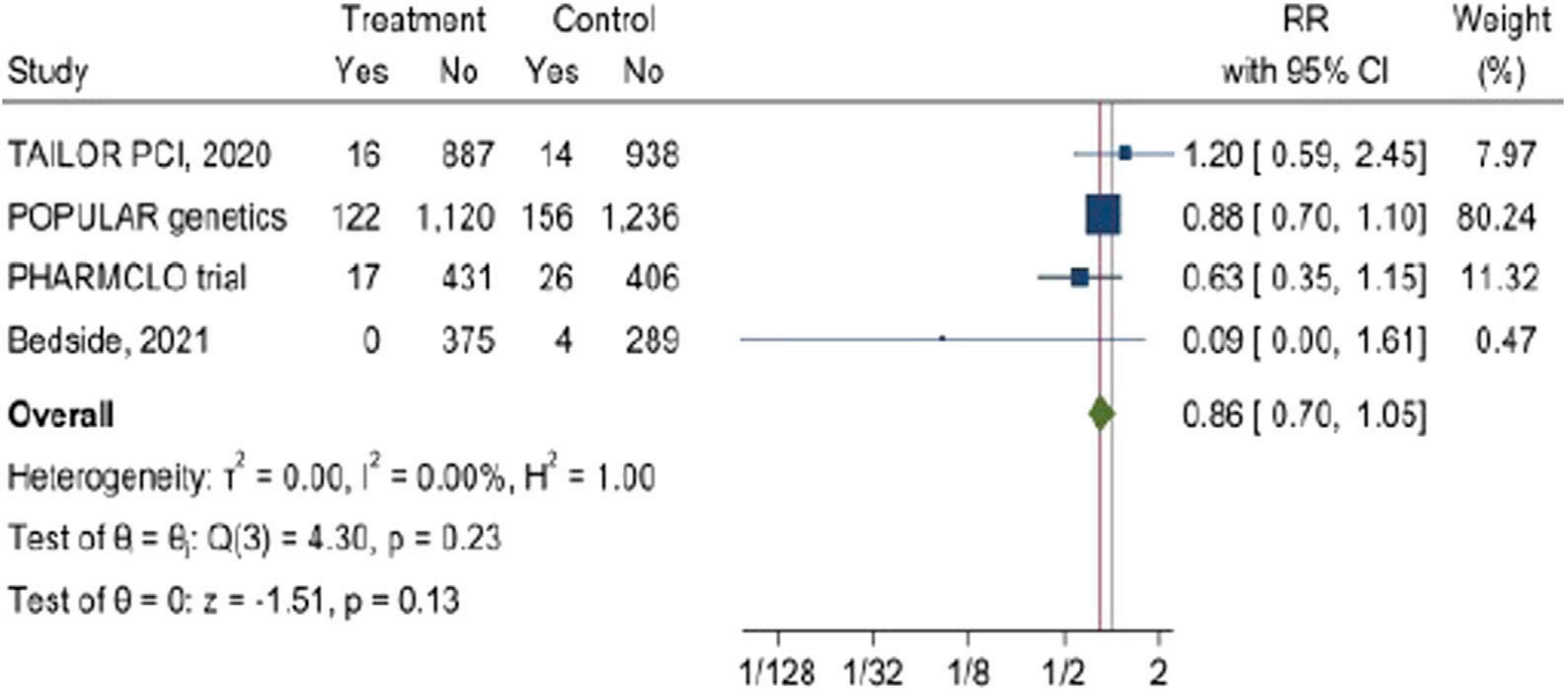
The impact of CYP2C19 point-of-care (POC) genotyping-guided antiplatelet therapy on Bleeding complications in patients undergoing percutaneous coronary intervention (PCI).

The pooled analysis from these four studies indicates that the RR ratio of bleeding is 0.86 (95% CI: 0.7, 1.05). The overall p-value of 0.13 from the z-test for the combined effect suggests that the reduction in the bleeding was not statistically significant. Importantly, there is no heterogenicity was observed among the investigated studies I^2^ = 0%. Figure 7.

### 3.9 The impact of CYP2C19 POC genetic testing on calculated composite outcome

**FIGURE 8:**
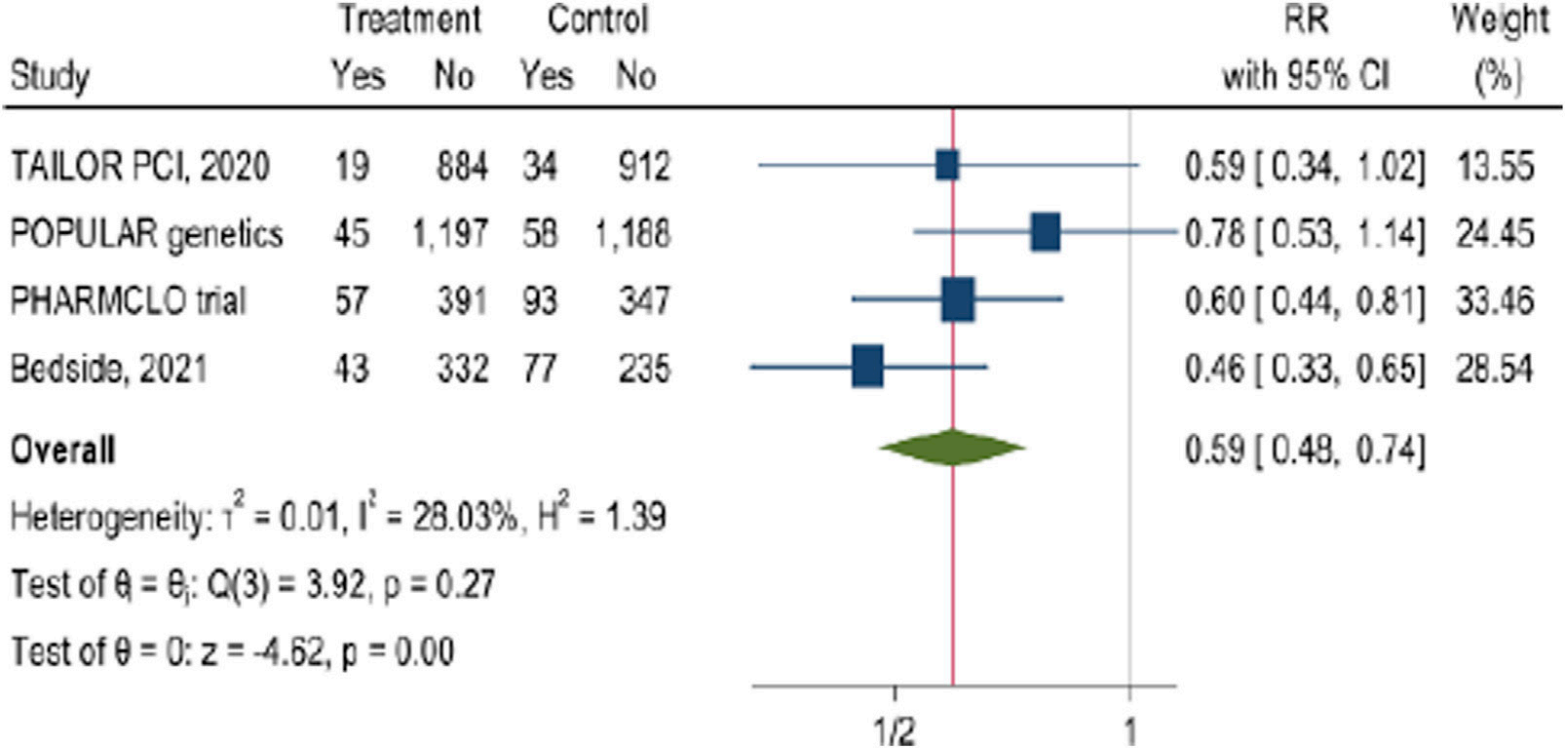
The impact of CYP2C19 point-of-care (POC) genotyping-guided antiplatelet therapy on calculated composite outcome in patients undergoing percutaneous coronary intervention (PCI).

The pooled analysis from these four studies (Figure 8) indicates was calculated for composite outcome in PCI patients which is typically includes MACE of cardiovascular death, myocardial infarction, stroke, In addition to stent thrombosis and bleeding complications. The results demonstrated by the forest plot showed that POC PGx-guided testing for the clopidogrel results in a significant decrease in the composite outcome (RR = 0.59, 95% CI: 0.48, 0.74). Although there was a considerable heterogenicity in the examined studies of I^2^ = 28.03% particularly in PHARMCLO trial which showed a robust benefit.

## 4 Discussion

PGx testing, particularly POC testing for the CYP2C19 gene, plays a critical role in managing patients undergoing PCI. Our systematic review and meta-analysis included four RCTs that compared POC genotyped versus non-genotyped guided strategies in patients treated with P2Y12 inhibitors for ACS or stable CAD undergoing PCI.

The introduction of POC genotyping technology, as demonstrated in the RAPID GENE study (Roberts et al., 2012), showcases its potential to rapidly guide personalized P2Y12 therapy following PCI. This approach is particularly beneficial for CYP2C19 LOF carriers ACS patients who exhibit a poor response to clopidogrel, enabling timely adjustments to alternative P2Y12 inhibitors for improved clinical outcomes (Pereira et al., 2024). However, a major challenge to its clinical implementation is the timing of genetic testing availability in relation to antiplatelet therapy initiation. Genotypic results are often not available at the time of the procedure and may take several days to return, by which time the treating cardiologist may no longer be involved in the patient’s care. A recent study by Massmann et al. (2024) highlighted the impact of preemptive genotyping on prescribing practices. Patients with CYP2C19 LOF alleles were less likely to be prescribed clopidogrel when readily available genotyping data was used. This emphasizes the need to integrate timely genetic testing into clinical workflows to improve therapeutic decision-making and enhance outcomes for PCI patients. In this analysis, we evaluate the impact of POC genotyping strategies on the overall burden of MACE in patients receiving antiplatelet therapy guided by genotyping versus those receiving standard care. This assessment is crucial for understanding how genotype-guided approaches influence clinical outcomes. By examining the role of POC genotyping in clinical decision-making, we can determine how timely and appropriate medication adjustments, informed by genetic testing, may reduce the long-term risk of adverse cardiovascular events. These findings further emphasize the importance of genotype-guided therapy in improving patient outcomes following PCI.

Our analysis showed a significant reduction in recurrent MI with a RR of 0.54 (95% CI: 0.38, 0.77), aligning with findings from previous studies by Dávila-Fajardo et al. 2019 and Cheng et al. 2023, which reported similar trends in genetically guided cohorts (Dávila-Fajardo et al., 2019; Cheng et al., 2023). However, the TAILOR-PCI trial, although valuable, was not sufficiently powered to demonstrate a clear superiority of genotype-guided therapy over standard clopidogrel treatment. The trial found a non-significant reduction in MI risk, highlighting the variability of outcomes across different populations and settings (Lopez et al., 2023).

Regarding the total composite MACE, including MI, stroke, stent thrombosis, or death, our analysis demonstrated a significant reduction in the composite MACE outcome with an RR of 0.59 (95% CI: 0.48, 0.74). This robust effect across studies supports the notion that POC PGx-guided testing can significantly enhance overall patient outcomes in the context of PCI. The role of immediate genetic testing results in shaping treatment strategies is paramount, underscoring the need for integrating PGx testing into clinical practice (Pereira et al., 2024). Notably, the consistent benefits across trials, particularly in PHARMCLO and Bedside (2021) (Notarangelo et al., 2018; Al-Rubaish et al., 2021), underscore the potential of CYP2C19 POC testing to inform timely adjustments in antiplatelet therapy, mitigating the risk of recurrent events. This rapid response capability is especially important in PCI settings, where the risk of recurrent MI is high, particularly in CYP2C19 LOF patients who do not respond adequately to standard therapy (Cargnin et al., 2023; Farag et al., 2019). The moderate heterogeneity observed for the composite outcome (I^2^ = 53.7%) may be partly attributed to ethnic and genetic variability across the study populations rather than methodological inconsistencies. Hardy–Weinberg equilibrium (HWE) analysis showed that most studies were in equilibrium, while minor deviations in a few datasets likely reflect natural population substructure or sampling variation rather than genotyping error.

In our pooled analysis comparing the outcomes of POC genotyping-guided strategies versus standard treatment, we observed a potential reduction in stroke, stent thrombosis, and bleeding rates, although these findings did not reach statistical significance. The lack of heterogeneity (I^2^ = 0%) suggests a consistent effect across studies, indicating that POC testing could assist clinicians in promptly modifying therapy to reduce stroke risk in high-risk populations. While not statistically significant, these results are clinically relevant, supporting the work of Roberts et al. (2012), who highlighted the importance of immediate access to genetic information for guiding treatment decisions and enabling early interventions to prevent adverse outcomes (Roberts et al., 2012). Additionally, we proposed that these findings suggest that POC testing could help balance the benefits and risks of antiplatelet therapy by allowing clinicians to make real-time adjustments based on individual patient profiles, thereby minimizing complications (Azzahhafi et al., 2023). Interestingly, some studies, such as those by van den Harmsze et al. and V Sibbing et al., reported significant associations between CYP2C19 variants and increased bleeding risk, highlighting the importance of comprehensive genetic assessments in clinical decision-making (Sibbing et al., 2010; Harmsze et al., 2012).

Our study emphasizes the role of CYP2C19 POC testing in guiding P2Y12 prescribing, an area not extensively studied before. POC testing is gaining popularity, with endorsements from the National Health Service (NHS) (Sinclair, 2023). The demonstrated benefits of POC genetic testing—such as rapid turnaround, accurate single SNP detection, cost-effectiveness, and seamless integration into electronic medical records—highlight its potential to support real-time, personalized prescribing decisions (Christodouleas et al., 2018; Abdel-Latif et al., 2024). These findings not only align with global initiatives aiming to advance precision medicine in cardiovascular care but also reinforce the strategic direction of the Qatar Precision Health Institute (QPHI) and national efforts in Qatar to implement PGx testing in guiding antiplatelet therapy (Abdel-Latif et al., 2024). Unlike other studies that presented pooled outcomes, which diluted results, our systematic review analyzed outcomes separately, providing clearer insights. Additionally, we included CYP2C19*17 in our analysis, which previous studies often overlooked, emphasizing its importance in bleeding risk assessment. By defining bleeding risk as minor and major and reporting the results separately, we provided a more nuanced understanding of the implications of PGx testing. The absence of asymmetry in the funnel plot suggests that publication bias is unlikely to have influenced our findings, although interpretation is limited by the small number of studies. Furthermore, the Q-Genie assessment indicated that the included genetic association studies were generally of high methodological quality, supporting the robustness of our pooled estimates. Our study’s limitations include the small number of included studies, which reduces the statistical power to explore heterogeneity in detail and precludes robust subgroup analyses for certain outcomes. Moreover, while we observed trends in several outcomes, not all reached statistical significance, underscoring the need for further research to confirm these findings.

In summary, our findings suggest that patients treated with clopidogrel based on POC CYP2C19 genotyping experience a lower risk of MACE, including myocardial infarction, stroke, stent thrombosis, and death, as well as reduced recurrent MI. This underscores the importance of genetic testing when prescribing clopidogrel for cardiovascular risk prevention. The impact of CYP2C19 POC genetic testing on PCI patient outcomes is substantial and the availability of timely results allows for immediate, informed decisions regarding medication prescriptions and adjustments, optimizing patient care and improving clinical outcomes.

## Data Availability

The original contributions presented in the study are included in the article/supplementary material, further inquiries can be directed to the corresponding author.
